# The President of the American Medical Association

**Published:** 1871-06

**Authors:** 


### The President of the American Medical Association.

The editor of the Richmond & Louisville *Medical Journal*, Prof. E. S. GAILLARD, M. D., of the Louisville Medical College, in his editorial for June, alleges that DAVID W. YANDALL, M. D., Prof. of Clinical Surgery in the University of Louisville, and *now*, *President of the American Medical Association*, and editor of the *American Practitioner*, is a QUACK!!!

At present, in the subject matter of the accusation, we have nothing to say, but as a journalist we simply announce the fact, and inform the profession that in Louisville, Ky., there is now raging a very active professional fire. If it should spread, it bids fair to *rage* considerably. Fire. of course, is not always an unmixed evil. It does, sometimes, clear up matters quicker and better than any other one of the elements. At this distance, and at present writing, it looks as though they would be obliged to send to Buffalo for "Babcock's Fire Extinguisher.

When this matter becomes reduced to proportions compatible with our columns, we may give a *resume* of the life and character of the President of the American Medical Association, meanwhile advise readers to suspend judgment.

